# Gastrointestinal Pathobionts in Pediatric Crohn's Disease Patients

**DOI:** 10.1155/2018/9203908

**Published:** 2018-07-17

**Authors:** Rachel V. Purcell, Nadeem O. Kaakoush, Hazel M. Mitchell, John F. Pearson, Jacqueline I. Keenan

**Affiliations:** ^1^Department of Surgery, University of Otago, Christchurch, New Zealand; ^2^School of Medical Sciences, University of New South Wales, Sydney, NSW, Australia; ^3^School of Biotechnology and Biomolecular Sciences, University of New South Wales, Sydney, NSW, Australia; ^4^Biostatistics and Computational Biology Unit, University of Otago, Christchurch, New Zealand

## Abstract

Crohn's disease (CD) is an inflammatory disease of the gastrointestinal tract, with a rising incidence worldwide, particularly in children. CD is thought to arise due to an immune response to environmental factors. The role of bacteria in CD has recently been highlighted, and here, we examine the prevalence of two bacterial species, enterotoxigenic *Bacteroides fragilis* (ETBF) and *Fusobacterium nucleatum*, implicated in gastrointestinal pathologies, in a pediatric CD cohort. Stool samples from 30 children with treatment-naïve CD and 30 age- and sex-matched controls were collected, and DNA was extracted. Quantitative PCR was used to determine the levels of ETBF and *F. nucleatum* in stool samples. Bacterial positivity and relative abundance were assessed between cases and controls and in relation to disease severity. No associations were found between colonization with ETBF and CD, or between colonization with either ETBF or *F. nucleatum* and disease severity or presence of *C. concisus*. However, a strong association was observed between positivity for *F. nucleatum* in the stool samples and the occurrence of CD in patients (25/30) as compared to controls (8/30) (*P*=0.003). *F. nucleatum* is more prevalent in the stool samples of pediatric CD patients, compared to healthy controls, and may have potential use as a biomarker of pediatric CD.

## 1. Background

Inflammatory bowel diseases (IBDs) are characterized by chronic inflammation in the gastrointestinal tract (GIT), and the two most commonly described forms of IBDs are Crohn's disease (CD) and ulcerative colitis (UC). CD is characterized by inflammatory lesions, which may be transmural, in any location in the GIT, with granulomas being a common feature. Although the etiology of CD is, as yet, unknown, there is evidence that dysregulated immune response to environmental triggers may lead to CD onset in genetically predisposed individuals.

CD affects around 3.2/1000 people in Europe and North America, and the incidence is rising, particularly in children. While previously the incidence of CD in children resident in nonindustrialized countries has been low, current evidence would suggest that, with industrialization, the incidence of CD is increasing [1].

CD is associated with a slightly lower life expectancy and considerable morbidity. Patients with long-standing CD located in the colon have a similar increased risk of colorectal cancer development as do those with UC [2, 3], and as a result, there has been intense interest in identifying potential etiological agents of the disease. The contribution of microbial dysbiosis to inflammation in the gut has recently been described, and several studies have identified changes in bacterial communities and particular species that are associated with CD. Decreased abundance of beneficial bacterial species from the Lachnospiraceae and Ruminococcaceae families and increased abundance of potential pathobionts, such as *Campylobacter* spp. and *Fusobacterium nucleatum*, have been identified using metagenomic and targeted studies of CD patients [4–7]. Both *C. concisus* and *F. nucleatum* are oral pathogens with epithelial cell adherence and invasive potential [4, 8–10]; certain strains of *C. concisus* produce exotoxin 9 and/or zonula occludens toxin (ZOT) [11]. Enterotoxigenic *Bacteroides fragilis* (ETBF) produces a zinc metalloprotease toxin called the *Bacteroides fragilis* toxin (Bft) that has been shown to induce colonic inflammation in mice [12, 13] and is associated with diarrheal disease and the development of colorectal cancer [14–16].

In this study, we investigate the abundance of ETBF and *F. nucleatum* in a cohort of pediatric Crohn's disease patients and age-matched controls. We also examined the association of these bacteria with the presence of previously determined *C. concisus* and its toxins, as well as with patient characteristics.

## 2. Materials and Methods

### 2.1. Study Subjects

Thirty children with Crohn's disease, diagnosed at Sydney Children's Hospital (Randwick, Australia), were recruited, in addition to 30 healthy controls. Stool samples were collected from the patients prior to treatment, and exclusion criteria included antibiotic or anti-inflammatory treatment in the four weeks prior to sample collection. This patient cohort has previously been described [6], and the levels of *C. concisus* and ZOT have previously been reported [17] in this cohort. Informed consent was obtained from all participants (or their parent/guardian), and the study was approved by the Research Ethics Committee of the University of New South Wales and the South East Sydney Area Health Service-Eastern Section, Sydney (Ethics nos. 03/163, 03/165, and 06/164).

### 2.2. Reference Strains

ETBF strain VPI 13784 [18] was generously supplied by Professor Cynthia Sears, Baltimore, USA, and was used as a reference strain in this study. This was cultured anaerobically on sheep blood agar (Fort Richard Laboratories). *F. nucleatum* was sourced from the American Type Culture Collection (ATCC) strain 25586. DNA was extracted from colonies recovered from the plates using the DNeasy Blood & Tissue Mini Kit (Qiagen, Hilden, Germany), as per the manufacturer's instructions. DNA extraction included digestion with Proteinase K for 3 hours at 56°C. The samples were stored at −20°C.

### 2.3. Primer Design

TaqMan primer/probe sets were designed to amplify regions of the *Bft* gene of ETBF and the *nusG* gene of *F. nucleatum*. Primer and probe sequences are shown in Table 1.

### 2.4. Quantitation of Genomic DNA in Bacterial Reference Strain Samples

Digital PCR (dPCR) was used to determine the concentration of bacterial genomes in each reference sample. The QuantStudio 3D Digital PCR System (Life Technologies) was used to carry out dPCR. Samples, consisting of 9 *µ*l of QuantStudio 3D Master Mix, 1.8 *µ*l of genomic DNA (25–35 ng/*µ*l of DNA), and 0.9 *µ*l of TaqMan primer/probe set (final concentrations of 900 nM/250 nM, resp.), in a final volume of 18 *µ*l, were loaded onto chips, and thermal cycling was carried out on a GeneAmp© PCR System 9700 thermocycler (Applied Biosystems), as previously described [21]. The chips were then loaded onto the QuantStudio™ 3D Digital PCR Instrument, and the endpoint fluorescence of each partition on the chips was measured using QuantStudio 3D Digital PCR software v3.0. Each sample was carried out in duplicate, and a no-template control was included with each run. Serial dilutions of each reference DNA sample were made to construct standard curves to determine levels of bacteria in test samples.

### 2.5. Quantitative PCR

DNA was extracted from fecal samples from CD cases and controls using the QIAamp DNA Stool Mini Kit (Qiagen, Hilden, Germany), as per the manufacturer's instructions. Levels of each bacterium were measured from genomic DNA samples using TaqMan probes on a LightCycler® 480 thermocycler (Roche Diagnostics, Indianapolis, IN, USA), as detailed in the Methods section of the study of Purcell et al. [22]. In brief, each reaction consisted of 25–35 ng of genomic DNA, 5 *µ*l of TaqMan Fast Advanced Master Mix (Applied Biosystems), and 0.5 *µ*l of TaqMan primer/probe (Thermo Fisher Scientific) in a 10 *µ*l reaction. Thermal cycling conditions were as follows: 1 cycle at 95°C for 10 min, followed by 50 cycles at 95°C for 10 sec and 60°C for 30 sec. All reactions were performed in triplicate.

### 2.6. Statistical Analysis

Fisher's exact tests, with 95% confidence intervals, were used to test for associations between Crohn's disease and the presence or absence of bacterial species. Pediatric CD activity index (PCDAI) values in the CD cohort were compared between patients tested negative and positive for each bacterial transcript, using *t*-tests, with Satterthwaite's adjustment for unequal variances and linear models, including covariates for age and gender. *P* values for association of CD and PCDAI values were adjusted for multiple testing using the Benjamini and Yekutieli method [23]. All analyses were carried out using R version 3.2.1 (Vienna, Austria).

## 3. Results

This study used a cohort of 30 newly diagnosed pediatric CD patients, of whom 20 were male; the patients ranged in age from 3 to 17.1 years (median = 11.4 years). Thirty healthy controls were also recruited, half of whom were male. The disease severity in patients was measured using the pediatric Crohn's disease activity index (PCDAI) and ranged from 7.5–70. Thirteen patients had mild disease (PCDAI < 30), while the remaining 17 patients had moderate-to-severe CD (PCDAI ≥ 30). All but one patient had evidence of colonic involvement, and as a result, we were unable to analyze bacterial abundance by disease location. Previous analysis of *C. concisus* and its toxins, exotoxin 9 and ZOT, showed that while the numbers of children tested positive for *C. concisus* were similar between cases and controls, the relative abundance of the bacteria and exotoxin 9 was higher in CD patients [6].

### 3.1. Association of Disease Severity with Presence or Absence of qPCR Products

**Figure 1:**
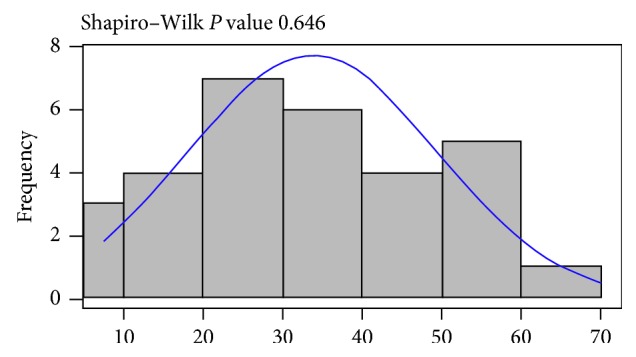
Histogram showing no evidence that disease severity, as measured by the pediatric Crohn's disease activity index (PCDAI), is not normally distributed.

Firstly, the distribution of PCDAI values was analyzed among the disease cohort using the Shapiro–Wilk test; there was no evidence that PCDAI was not normally distributed among CD patients in the cohort (*P*=0.646; Figure 1). No evidence of an association between CD severity, as measured by the PCDAI, and the presence or relative abundance of any bacterial transcripts or toxins was observed (all adjusted *P* values > 0.1; Table 2). This is similar to findings reported with *C. concisus*.

### 3.2. Association of CD and Bacterial Transcripts in Fecal Samples

Quantitative PCR revealed that two patients and three controls were positive for ETBF, while 25 patients with CD were positive for *F. nucleatum*, compared with only eight controls. Analysis of associations between bacterial transcript positivity and the presence of CD, using Fisher's exact test, found a strong association between *F. nucleatum* positivity in stool samples and the occurrence of CD. The *P* value was 0.00002, and when adjusted for age and gender, it was 0.0003. There was no significant difference or trend in the relative abundance of *F. nucleatum* between cases and controls.

## 4. Discussion

In our study, we have investigated the prevalence and abundance of specific bacterial species in a cohort of pediatric Crohn's disease patients and matched controls. The study was conducted on fecal stool samples from newly diagnosed patients prior to treatment, and information on disease severity and the abundance of *C. concisus* and its toxins was known prior to the study being carried out. Microbial dysbiosis, involving a reduction in fecal bacterial diversity, has been widely reported in gastrointestinal diseases, including CD, ulcerative colitis, and colorectal cancer (CRC) [24–27], and there has been much focus on identifying particular bacterial species that may play a role in disease pathogenesis, as therapeutic targets and biomarkers of disease.

*Bacteroides fragilis* is a common gut commensal in adults, although the rate of carriage is not well characterized in children. Enterotoxigenic strains of *B. fragilis* (ETBF) secrete a proinflammatory metalloprotease toxin and have been associated with diarrheal disease in both children and adults. ETBF has also been proposed as a potential initiator of CRC via E-cadherin cleavage and NF-kB activation, as demonstrated in *in vitro* and animal studies and increased carriage of the bacterium in stool samples of CRC patients compared to controls [16, 28]. In contrast, the evidence linking ETBF to IBD is limited, although a recent publication has demonstrated increased carriage of ETBF in patients with UC, compared to controls [29]. In this study, we found no association between the presence or abundance of toxigenic strains of *B. fragilis* in the stool samples and the occurrence of CD in patients as compared to healthy controls.

Intestinal colonization with oral bacteria has recently been implicated in inflammatory bowel diseases [5], with toxin-producing *Campylobacter* species being associated with pediatric CD as compared to healthy controls in this cohort and others [6, 17, 30].

*Fusobacterium nucleatum* is another prevalent oral bacterium found in extraoral diseases and has the ability to adhere to and invade epithelial cells via its surface adhesin molecule, FadA. Strains of *F. nucleatum* isolated from IBD have been shown to be significantly more invasive than strains from healthy tissue or controls [4], and colonization of epithelial cells by *F. nucleatum* is associated with an inflammatory response [31–33]. Although causality has yet to be established, increased abundance of this bacterium has frequently been associated with CRC [19, 34, 35] and IBD, including CD, a known risk factor for CRC. Two separate microbiome studies of pediatric CD (*n*=19 patients in both studies) found enrichment of *Fusobacterium* sp. in fecal samples of patients compared to healthy controls. The study by Shaw et al. found that *Fusobacterium* sp. were more abundant in cases compared to controls and between nonresponders and responders to immunomodulatory treatment [36], while the study by Kaakoush et al. [17] found *Fusobacterium* sp. in 8/19 CD patients compared to 1/19 controls. Although these studies used a 16S rRNA sequencing approach that often does not allow for discrimination to the species level, the findings are similar to those of our direct bacterial species testing using qPCR in this study, where we found a strong association of *F. nucleatum* with CD as compared to control samples. *F. nucleatum* was detected in 83.3% of CD cases compared to 26.7% of controls, with similar abundance of the bacterium in samples from the two groups.

Testing for *F. nucleatum* in fecal samples, in combination with fecal immunochemical testing (FIT), improves detection of adenomas and CRC compared to FIT alone [37, 38], and our findings indicate that this oral pathogen may also prove to be a useful tool in noninvasive fecal testing for CD. A study by Dharmani et al. has reported that different strains of *F. nucleatum* vary in relation to their inflammatory and invasive potential [10]. This strain variance should be taken into account in future clinical studies.

Future directions would include validation of different strains of *F. nucleatum* as markers of disease in a larger independent cohort and testing the utility of *F. nucleatum* as a marker of disease activity. *F. nucleatum* has recently been linked to chemoresistance in colorectal cancer [39], and its influence on response to therapy may also have relevance in the treatment of CD. The strong association of the bacteria with CD patients compared to controls also provides direction for future functional studies on the pathogenesis and progression of CD using *in vitro* and *in vivo* models.

## 5. Conclusions

*F. nucleatum* is more prevalent in the stool samples of pediatric Crohn's disease patients, compared to healthy controls, and may have potential use as a biomarker of pediatric CD.

## Figures and Tables

**Table 1:** Primers and probes used for quantitative PCR.

Gene	Sequence 5′ → 3′	Reference
*F. nucleatum nusG*		[19]
Forward primer	CAACCATTACTTTAACTCTACCATGTTCA	
Reverse primer	GTTGACTTTACAGAAGGAGATTATGTAAAAATC	
Probe	TCAGCAACTTGTCCTTCTTGATCTTTAAATGAACC	

*Bft*		[20]
Forward primer	GGATAAGCGTACTAAAATACAGCTGGAT	
Reverse primer	CTGCGAACTCATCTCCCAGTATAAA	
Probe	CAGACGGACATTCTC	

**Table 2:** Association of PCDAI and transcripts in the gut.

	Negative mean	Positive mean	Negative SE	Positive SE	*P* value	*P* value (adj.)
Cc	27.500	35.292	3.416	3.375	0.124	0.345
ZOT	29.375	35.318	4.324	3.505	0.301	0.749
ETBF	33.685	34.167	3.126	3.333	0.919	0.319
Fn	35.500	33.380	7.045	3.140	0.793	0.768

Cc, *Campylobacter concisus*; ZOT, zonula occludens toxin; ETBF, enterotoxigenic *Bacteroides fragilis*; Fn, *Fusobacterium nucleatum*; SE, standard error; adj., adjusted for age and gender.

## Data Availability

The qPCR data used to support the findings of this study are available from the corresponding author upon request.
